# New Mode of Operating in Hare-Lip

**Published:** 1855-07

**Authors:** 


### ARTICLE XI.

### *New Mode of Operating in Hare-lip.* By Mr. Erichsen.

Mr. Erichsen recently opened the Summer Session at University College, London, by a clinical lecture on Hare-lip, from which we give extracts.

Gentlemen.—The College of Surgeons, you are aware, have extended their curriculum, and now require you to attend clinical lectures for the summer for the first time. In compliance with this regulation, we are met here to-day. The subject I have chosen is one in which you may be engaged early in practice—hare-lip; apparently so very simple, but so much the more it is necessary you should all quite understand it. We have lately employed the simple suture in the treatment of hare-lip, both in single and double hare-lip, with the very best results; thus dispensing with the use of the more cumbersome means of pins and the twisted suture. The advantage of this method over the old one is, that it is more simple, attended by less scarring to the face, and this being essentially an operation of *convenance*, as the French term it, or an operation to remedy a deformity, it cannot be done too accurately or nicely. We shall have an opportunity of showing you some cases, and here are drawings, both before and after operation, of some interest and practical importance. After paring the edges of the fissure in the usual way, if the cleft be single, as in this drawing, we introduce two points of interrupted suture, of the ordinary twist, well waxed, the lower one being carried through the lip at the junction of the *prolabium* and integument, so as to include and compress the labial, or rather coronary artery of the upper lip, a branch you remember, of the facial. The upper ligature next, midway between this and the nose. We next introduee one point of very fine suture just *inside* the lip, through the mucous membrane; the lip is then supported by two narrow strips of plaster, and on the third or fourth day, both may be removed.

In double hare-lip, where the tension is very great, more epecially if there be a very considerable intermaxillary fossa, I think you may, in some subjects, press down the piece of maxillary bone a little, and afterwards we use this instrument—a sort of double spring, coming around the head and pressing on the upper lip, relieving the tension on the parts and doing, in another and less disfiguring way, what the long pins did in the old operation. This instrument has a little history of itself, having been invented, as you are aware, by a Mr. Hainsby, who had a child of his own with hare-lip, where the old operation by pins failed, from the child crying and dragging the parts asunder. I think the pins act, so to speak, as setons while they are in the lip; and always leave little spots on the lip where suppuration has been going on. This is to be avoided; and in practice you will find a nicely-done operation of hare-lip, or one clumsily done, is always a sort of advertisement of your skill. Mothers, too, are so fond of their children that an ugly scar or a junction of the parts quite linear, and not to be noticed, makes all the difference in the world.

The following cases which I will read to you out of the book, with the engravings and sketches, and two of the children which happen to be here, just over the operation, will illustrate the point further. I would only say in addition, it is always advisable to do the operation early—before the period of dentition; at this time, too, I think the plastic elements of the infant, just released from the mother's womb, are in greater activity.

Mr. Erichsen then read the following cases from the books of University College Hospital:—

*Case* 1.—An infant six days old, with single hare-lip, admitted under Mr. Erichsen, October 11, 1854; the edges of the fissure were pared, and two points of suture introduced through the lip, with one on the mucous membrane. The first suture was taken out on the third day, the next on the fourth, and the lip was firmly united with remarkably little appearance of scarring by the sixth day.

*Case* 2.—A child ten months old, teething, and excessively irritable, admitted under Mr. Erichsen on the 18th of April, with double hare-lip, wide fissure of the palate, and very prominent intermaxillary projections—presenting altogether, from the age and irritability of the child, and the amount of deformity, a very unfavorable case. Mr. Erichsen, after bending down the intermaxillary projection, and paring the edges of the cleft in the usual way, brought them together with two sutures in either side, and one in the mucous membrane. "Hainsby's cheek-compressor" was then applied, and all tension, which would otherwise have been very great, taken off the sutures. These came away on the third day, and solid union of the lip resulted. The child was kept in the hospital for a fortnight, during which time it wore the cheek-compressor, lest during fits of violent screaming and convulsions to which it was subject, the lines of union should be weakened.

*Case* 3.—A child four years of age, with single hare-lip, admitted April 25th. In this case the operation was done in the same simple way as in the first. The cheek-compressor being required, the sutures were removed on the third and fourth days, and the child discharged cured, with a very perfect lip indeed, at the end of the week.

These cases, occurring at very different ages, and in the second instance under the most unfavorable circumstances, illustrate the advantages of the simple suture in the treatment of this deformity, aided when there is any appearance of tension about the parts by the cheek-compressor, which at once takes off all traction from the lip.—*Dub. Med. Press.*

